# The bright side of reactive oxygen species: lifespan extension without cellular demise

**DOI:** 10.15761/JTS.1000138

**Published:** 2016-04-28

**Authors:** Kenneth Maiese

**Affiliations:** Cellular and Molecular Signaling, Newark, New Jersey 07101, USA

**Keywords:** apoptosis, autophagy, cell longevity, forkhead transcription factors, mechanistic target of rapamycin (mTOR), mitochondria, nicotinamidases, oxidative stress, programmed cell death, reactive oxygen species, sirtuins, Wnt signaling

## Abstract

Oxidative stress and the generation of reactive oxygen species (ROS) can lead to mitochondrial dysfunction, DNA damage, protein misfolding, programmed cell death with apoptosis and autophagy, and the promotion of aging –dependent processes. Mitochondria control the processing of redox energy that yields adenosine triphosphate (ATP) through the oxidation of glucose, pyruvate, and nicotinamide adenine dinucleotide. Ultimately, the generation of ROS occurs with the aerobic production of ATP. Although reduced levels of ROS may lead to tolerance against metabolic, mechanical, and oxidative stressors and the generation of brief periods of ROS during ischemia-reperfusion models may limit cellular injury, under most circumstances ROS and mitochondrial dysfunction can lead to apoptotic caspase activation and autophagy induction that can result in cellular demise. Yet, new work suggests that ROS generation may have a positive impact through respiratory complex I reverse electron transport that can extend lifespan. Such mechanisms may bring new insight into clinically relevant disorders that are linked to cellular senescence and aging of the body’s system. Further investigation of the potential “bright side” of ROS and mitochondrial respiration is necessary to target specific pathways, such as the mechanistic target of rapamycin, nicotinamidases, sirtuins, mRNA decoupling and protein expression, and Wnt signaling, that can impact oxidative stress-ROS mechanisms to extend lifespan and eliminate disease onset.

## Increased reactive oxygen species production through reverse electron transport may extend lifespan and prevent programmed cell death

Reactive oxygen species (ROS) are generated during oxidative stress that include nitrogen based free radical species, such as nitric oxide and peroxynitrite, and oxygen derivatives involving superoxide free radicals, hydrogen peroxide, and singlet oxygen [1-3]. Mitochondria lead to the generation of ROS. Mitochondria yield adenosine triphosphate (ATP) through the oxidation of glucose, pyruvate, and nicotinamide adenine dinucleotide (NAD^+^) that exist in the cytosol. In the tricarboxylic acid cycle, NAD^+^ and flavin adenine dinucleotide (FAD) are reduced to NADH and FADH_2_. The redox energy from NADH and FADH_2_ is transferred to oxygen through the electron transport chain. This process allows protons to be transferred from respiratory complexes I, III, and IV in the inner membrane to the intermembrane space with a subsequent proton gradient that is formed across the inner membrane. Complex V (ATP synthase) subsequently accumulates the energy from this gradient to produce ATP from adenosine diphosphate (ADP) and inorganic phosphate (P_i_). With the aerobic production of ATP, the generation of ROS occurs [4].

A fine balance appears necessary for the generation of ROS to limit cell injury and extend lifespan. For example, moderate levels of ROS may be required for the tolerance against metabolic, mechanical, and oxidative stressors [5] and the generation of brief periods of ROS during ischemia-reperfusion models may limit cellular injury [6,7] through several different pathways such as those that involve the mechanistic target of rapamycin (mTOR) [8] or Wnt signaling [9,10]. Yet, at increased levels, ROS through oxidative stress can result in mitochondrial and other organelle injury, DNA damage, protein misfolding, cell demise, and the promotion of aging [11]. The depletion of NAD^+^ has been associated with aging and the maintenance of adequate NAD^+^ stores has been linked to a reduction in the aging process and increased resistance to oxidative stress [12]. In addition, agents such as nicotinamide may reduce ROS and prevent cellular senescence [13,14]. At high levels of ROS generation, mitochondrial dysfunction and oxidative stress also can result in the induction of apoptotic pathways [11,15-18]. Mitochondrial dysfunction results in the opening of the mitochondrial membrane permeability transition pore, release of cytochrome c, and apoptotic caspase activation [19-21]. Other pathways of programmed cell death also may be involved during oxidative stress and mitochondrial dysfunction [22,23]. Autophagy can impair endothelial progenitor cells, and lead to mitochondrial oxidative and endoplasmic reticulum stress [15,24]. However, autophagy also may be necessary for the removal of misfolded proteins and to eliminate non-functioning mitochondria [25] that has been shown to maintain β-cell function and prevent the onset of diabetes mellitus [26].

Interestingly, new work suggests that ROS may be necessary for the promotion of extended lifespan [27]. Although the work supports prior studies that increased ROS can lead to injury and reduce lifespan, the study also illustrates that ROS production with reduced ubiquinone and possibly through respiratory complex I reverse electron transport can extend lifespan in *Drosophila*. The authors suggest that an intact respiratory complex I may be required in this model as compared to other studies that can reverse oxidative damage with blockade of respiratory complex I [28].

There are a number of cell signaling pathways that may be tied to these mitochondrial processes that extend lifespan and control the aging process. For example, increased decoupling of mRNA and protein expression can affect mTOR signaling and aging –dependent changes [29]. Hormones such as melatonin can oversee pathways of insulin-like growth factor 1 to increase lifespan [30]. Modulation of of nicotinamidases and sirtuin pathways also are involved in lifespan extension [31-34]. Down-regulation of mTOR pathways [35-38] as well as modulating forkhead transcription factors [39-42] may be another avenue to control cell senescence, extend lifespan, and modulate the process of aging. Each of these mechanisms are clinically relevant and impact the aging process throughout the body such as the musculoskeletal system [43] and the endocrine system [44]. Further investigation is certainly warranted to target the potentially beneficial aspects of ROS generation through mitochondrial respiration to modulate the aging process of organisms and, in turn, hopefully extend lifespan and reduce disease onset.
